# Active ingredient and mechanistic analysis of traditional Chinese medicine formulas for the prevention and treatment of COVID-19: Insights from bioinformatics and in vitro experiments

**DOI:** 10.1097/MD.0000000000036238

**Published:** 2023-12-01

**Authors:** Jiakai Yang, Qianqian Zhuang, Chi Zhang, Xinli Liu

**Affiliations:** a Department of Biological Engineering, Qilu University of Technology, Jinan, China.

**Keywords:** COVID-19, in vitro ex, molecular docking, network pharmacology, prevention and treatment

## Abstract

Coronavirus disease 2019 (COVID-19) is an acute infectious disease caused by a novel coronavirus. Traditional Chinese medicine (TCM) has been proven to have a potential curative effect on COVID-19. This study preliminarily analyzed the existing TCM prescription’s key components and action mechanisms for preventing and treating COVID-19 using bioinformatic and experimental methods. Association and clustering analysis reveals that the “HQ + FF + BZ” drug combination had a strong correlation and confidence in 93 TCM prescriptions and may affect the progression of COVID-19 through inflammatory pathways such as the TNF signaling pathway. Further molecular docking revealed that quercetin has a higher affinity for IL6 and IL10 in the TNF signaling pathway associated with COVID-19. In vitro experiments demonstrated that quercetin could effectively reduce the levels of the inflammatory factor IL-6 and increase the anti-inflammatory factor IL-10, alleviating inflammation impact on cells. Our results provide a new understanding of the molecular mechanism of TCM prevention and treatment of COVID-19, which is helpful to the development of new diagnosis and treatment schemes for COVID-19.

## 1. Introduction

Novel coronavirus pneumonia emerged at the end of 2019 and was named coronavirus disease 2019 (COVID-19) by the World Health Organization (WHO). It is an acute infectious disease primarily characterized by fever and cough, caused by a novel coronavirus.^[1,2]^ According to WHO data released as of December 23, 2022, there have been approximately 651,918,402 confirmed cases of COVID-19 globally, with 6656,601 reported deaths.^[3]^ Severe acute respiratory syndrome coronavirus 2 (SARS-CoV-2) mainly spreads through respiratory droplets, with minor transmission routes including indirect contact and fecal-oral transmission.^[4]^ SARS-CoV-2 initially binds to angiotensin-converting enzyme 2 (ACE2) receptors on the cell membranes of oral, nasal, and alveolar epithelial cells and enters the cells through the activation of serine protease (TMPRSS2).^[5–7]^ SARS-CoV-2 exhibits high transmissibility and variability, and its variant strain, Omicron, has shown enhanced transmissibility, reduced pathogenicity, and increased immune evasion. Although its severity rate is low, it has led to a broader range of infections, increased hospitalization rates among elderly patients, and an increased burden on public health.^[8,9]^

In Western medicine, the treatment of COVID-19 is divided into macromolecular drugs and small molecular drugs. Small molecular drugs, such as Remdesivir and Paxlovid, achieve therapeutic effects by blocking the replication and transcription of the novel coronavirus RNA.^[10,11]^ Macromolecular drugs include monoclonal antibody drugs targeting viral host receptors and proteins related to viral replication and transcription. However, multiple antibody drugs have been proven ineffective against the Omicron variant strain.^[10]^

COVID-19 belongs to the category of “plague” in TCM, and TCM has played a crucial role in treating plague since ancient times. TCM treatment plans were incorporated as early as the “Diagnosis and Treatment Protocol for Novel Coronavirus Pneumonia (Second Edition)” in China. In the TCM treatment of COVID-19, Liu et al^[12]^ found that Lianhua Qingwen Granules had an excellent therapeutic effect against the Omicron variant strain. Li et al^[13]^ showed that Xuanfei Baidu Decoction had better therapeutic effects than Western medicine and conventional treatment in severe COVID-19 patients, improving inflammatory responses and clinical symptoms. Wang et al^[14]^ demonstrated that Qingfei Paidu Decoction, in combination with conventional Western medicine treatment, effectively improved clinical symptoms in elderly COVID-19 patients. In addition, TCM emphasizes disease prevention, highlighting the importance of treating before illness and prioritizing prevention over treatment. On the other hand, TCM interventions are used for the already affected population to prevent further disease progression, reflecting the critical concept of TCM in disease prevention and treatment.^[15]^ Considering differences in climatic conditions, population characteristics, and geographical factors among provinces and cities, different COVID-19 prevention and treatment regimens have been introduced across China. Research by Ma et al^[16]^ showed that the Hunan COVID-19 Prevention Formula 2 increased the spleen index of tested mice and reduced the expression of inflammatory factors IL6 and TNF-α in experimental influenza virus infection, improving the immune function of mice. The new crown control programs introduced in different regions vary. Therefore, identifying the common groupings among them, understanding their mechanisms in controlling COVID-19, and discovering the critical active ingredients can play an essential role in further developing COVID-19 control programs.

In this study, we aim to analyze the patterns of prevention and treatment formulations for COVID-19 from a bioinformatics perspective using data mining, network pharmacology, and molecular docking methods. The study aims to explore the underlying mechanisms of these formulations and provide references for understanding the mechanism of prevention and treatment formulations and for developing future COVID-19 prevention and treatment strategies.

## 2. Materials and methods

### 2.1. Acquisition and processing of COVID-19 prevention and treatment formulas

The prescriptions used in this study were derived from official TCM prevention and treatment regimens published in various provinces and cities in China in mid-2021, such as Henan, Heilongjiang, and Sichuan, excluding non-oral formulations and duplicated formulas. The sorted medicinal ingredients of the prescriptions were entered into a table, and the recorded data were audited. The names of each TCM were standardized according to the “Pharmacopoeia of the People’s Republic of China” and “Chinese Medicinal Dictionary” to ensure the accuracy of the drug names. Each TCM was listed as a variable, and Excel functions were used to perform binary classification on the terms of the prescriptions. If a corresponding TCM name was present in the drug, it was recorded as “1”; otherwise, it was recorded as “0.” Thus, a table was established in the form of binary variables for the prescription names and their corresponding included TCMs.

### 2.2. Data mining of COVID-19 prevention and treatment formulas

The prescriptions meeting the criteria were initially processed using WPS Office 11.1.0 to count the frequency of use of each TCM, and the drugs were organized into a table with binary variables. IBM SPSS Statistics 26 was used to conduct a hierarchical cluster analysis of the top thirty TCMs with the most considerable frequency using the Pearson correlation algorithm, and a cluster dendrogram was generated to determine the clustering of TCMs based on distance. IBM SPSS Modeler 18.0 was then used to conduct association analysis based on the Apriori algorithm on the table of binary variables. Key drug combinations were obtained by combining hierarchical clustering and association analysis.

### 2.3. Selection of effective compounds and targets of core drug combinations

The compounds included in the critical drug combinations and their corresponding target information were obtained from databases. Blends with oral bioavailability ≥ 30% or drug-likeness ≥ 0.18 were selected from databases, including TCMSP (https://tcmsp-e.com/tcmsp.php),^[17]^ ETCM (http://www.tcmip.cn/ETCM/),^[18]^ and BATMAN-TCM (http://bionet.ncpsb.org.cn/batman-tcm/).^[19]^ Subsequently, the compounds were matched with their corresponding target information.

### 2.4. COVID-19 target screening

The DrugBank database (https://go.drugbank.com/),^[20]^ Genecard database (www.genecards.org),^[21]^ Therapeutic Target Database (https://db.idrblab.net/ttd/),^[22]^ and DisGeNET database (https://www.disgenet.org/)^[23]^ were searched using COVID-19 or related keywords. Targets with a relevance score ≥ 10 from the Genecard database and with a literature count ≥ 5 from the DisGeNET database were selected. All obtained genes were unified using the UniProt database (http://www.uniprot.org/)^[24]^ to get the corresponding gene symbols, and all targets obtained were merged and deduplicated. The Venn diagram was drawn using the R package “Venn” to obtain common targets between the effective compounds and the screened COVID-19 targets.

### 2.5. Construction and optimization of the network of TCM-effective compounds-targets

Since the critical drug combinations have numerous corresponding compounds and targets, constructing a network diagram that includes all of them would be complex and challenging to obtain the desired information. Therefore, in this network diagram, only the common targets of COVID-19 and effective compounds were selected, along with the corresponding compounds. Cytoscape v3.9.0^[25]^ was used to draw the TCM-effective compounds-targets network, adjusting each node’s transparency, size, and color based on its degree value.

### 2.6. Construction of protein-protein interaction (PPI) network

A PPI network diagram was constructed to understand further the interactions between the critical drug combinations and the targets for COVID-19 prevention and treatment. The STRING website was used to construct the interaction network of the 72 common targets, which were then divided into 3 clusters using K-means clustering. The data were subsequently imported into Cytoscape software for analysis to identify critical genes in the network.

### 2.7. Differential gene expression analysis in GEO database

The GEO database (https://www.ncbi.nlm.nih.gov/geo/)^[26]^ contains various public clinical data for different diseases, providing gene expression profiles of multiple illnesses. The GSE164805 gene chip was obtained from the GEO database, including gene expression profiles of 5 normal individuals, 5 mild COVID-19 patients, and 5 severe COVID-19 patients. After data processing, gene expression heatmaps and volcano plots were generated using R software.

### 2.8. GO and KEGG analysis

The DAVID database (https://david.ncifcrf.gov/),^[27,28]^ a widely-used website for GO and KEGG analysis, was used to obtain up-to-date data. GO analysis includes Biological Process (BP), Molecular Function (MF), and Cellular Component (CC). GO analysis helps identify GO terms significantly enriched in common and critical genes, providing insights into their functional roles. KEGG analysis shows enriched pathways, facilitating the understanding of their mechanisms. The data obtained from GO and KEGG analyses were plotted using R packages and Origin2022 software. The R package Pathview was used to annotate the core targets selected from the screened common targets on the corresponding pathways, and gene upregulation and downregulation were highlighted in different colors further to identify the core targets among the key targets.

### 2.9. Molecular docking

The original protein structures of the core targets were obtained in PDB format from the PDB database (https://www.rcsb.org/).^[29]^ Pymol 2.4.0 software removed water molecules and redundant ligands and saved the processed protein structures. The structures of small molecule effective compounds were obtained from the PubChem database (https://pubchem.ncbi.nlm.nih.gov/). The processed protein and effective compound structures were subjected to semi-flexible docking using Autodock v1.5.6, and the docking results were analyzed using the PILP website (https://plip-tool.biotec.tu-dresden.de/plip-web/plip/index).^[30]^

During Autodock docking, the grid box was limited to the known active site. If no relevant reports were available, predictions were made using the DeepSite online website (https://playmolecule.com/deepsite/).^[31]^ The conformation with the lowest binding energy within the active region was selected from the docking results of each protein-compound pair for further analysis. The final images were optimized using Pymol software, and the protein-ligand interactions were visualized using Ligplot + v2.2.^[32]^

### 2.10. In vitro cell experiments

#### 2.10.1. Materials and instruments.

A549 alveolar epithelial cells were obtained from the Analysis and Testing Center of Qilu University of Technology. TNF-α (Batch No. 10602-HNAE, purity ≥ 95%, purchased from Beijing Yiqiao Shenzhou Biotechnology Co., Ltd., Beijing Economic and Technological Development Area, Beijing, China), Quercetin (Batch No. S25567, purity ≥ 97%, purchased from Shanghai Yuan Ye Biological Technology Co., Ltd., Songjiang District, Shanghai, China), Human interleukin 6 (IL-6) ELISA kit (Batch No. JM-03204H2), and Human interleukin 10 (IL-10) ELISA kit (Batch No. JM-03204H2) were purchased from Jiangsu Jingmei Technology Co., Ltd (Yancheng City, Jiangsu Province, China). High-glucose DMEM culture medium (Batch No. 8121731, purchased from Thermo Fisher Scientific (China) Co., Ltd., Pudong New District, Shanghai, China), fetal bovine serum (Batch No. 21110704, purchased from Zhejiang Tianhang Biotechnology Co., Ltd., Luoshe Town, Deqing County, Zhejiang Province, China), and Mycoplasma elimination reagent (Batch No. AM2247K1, purchased from Shanghai Saina Biological Technology Co., Ltd., Changning District, Shanghai, China) were also used. Other instruments included a carbon dioxide incubator (Jinan Xinbeixi Biological Technology Co., Ltd., Changning District, Shanghai, China), an inverted microscope (Olympus (China) Co., Ltd., Xuhui District, Shanghai, China), an electric constant temperature incubator (Shanghai Jinghong Experimental Equipment Co., Ltd., Pudong New District, Shanghai, China), an enzyme immunoassay analyzer (American Biotec Instruments Co., Ltd., Vermont, USA), and a clean bench (Shanghai Zhicheng Analysis Instrument Manufacturing Co., Ltd., Fengxian District, Shanghai).

#### 2.10.2. Experimental procedures.

A549 alveolar epithelial cells were cultured in a carbon dioxide incubator in a high-glucose DMEM medium containing 10% fetal bovine serum and antibiotics (100 U/mL penicillin and 100 U/mL streptomycin). The culture medium was changed every other day, and the cells were passaged at a 1:3 ratio every 3 days. The Mycoplasma elimination reagent was added after each medium change and passage to prevent contamination. When the cells reached the third passage and were stable, they were used for the experiments. 2 × 10^5^ A549 cells were evenly seeded in each well of a 24-well plate and incubated for 24 hours before treatment. The experimental groups were as follows: Negative control group: A549 cells cultured in complete medium; Positive control group: A549 cells stimulated with TNF-α (10 ng/mL) in complete medium; Prevention and treatment groups: Three groups treated with different concentrations of quercetin and the same concentration of TNF-α as the positive control group, with 4 replicate wells per group. The cells were incubated in different groups for 24 hours in a carbon dioxide incubator. After incubation, the supernatant of each well was collected, aliquoted, and stored at −80°C for subsequent analysis. The secretion levels of IL-6 and IL-10 in the culture supernatant were determined according to the ELISA kit instructions. The experimental results were analyzed using SPSS software and visualized using Origin 2018.

## 3. Results

### 3.1. Data mining of COVID-19 prevention and treatment formulas

#### 3.1.1. Frequency analysis of COVID-19 prevention and treatment formulas.

In this analysis, a total of 95 prescriptions containing 91 different traditional Chinese medicines for COVID-19 prevention and treatment from various regions were included. The total frequency of drug occurrences was 714. Among them, 17 drugs had a frequency greater than 10, with 491 events (approximately 69% of the total drug occurrences). These drugs included Huangqi, Guanghuoxiang, Gancao, Fangfeng, Baishu, and others (Table 1). The main types of traditional Chinese medicines in the prevention and treatment formulas were Qi Reinforcing Drugs. These 17 drugs were selected as high-frequency for subsequent association and cluster analyses.

**Table 1:** High-frequency drugs and their types in COVID-19 prevention and treatment formulas (≥10 times).

Number	Herb pinyin	Latin name	Herb types	Frequency
1	Huangqi (HQ)	Hedysarum Multijugum Maxim.	Qi Reinforcing Drugs	54
2	Guanghuoxiang (GHX)	Pogostemon Cablin (Blanco) Benth.	Dampness Removing Drugs	50
3	Gancao (GC)	licorice	Qi Reinforcing Drugs	49
4	Fangfeng (FF)	Saposhnikoviae Radix	Pungent-Warm Exterior-Releasing Medicinal	44
5	Lugen (LG)	Phragmitis Rhizoma	Fire Purging Drugs	43
6	Jinyinhua (JYH)	Lonicerae Japonicae Flos	Antipyretic Detoxicate Drugs	34
7	Baizhu (BZ)	Atractylodes Macrocephala Koidz.	Qi Reinforcing Drugs	34
8	Chenpi (CP)	Citrus Reticulata	Qi Regulating Drugs	29
9	Lianqiao (LQ)	Forsythiae Fructus	Antipyretic Detoxicate Drugs	27
10	Cangzhu (CS)	Atractylodes Lancea (Thunb.)Dc.	Dampness Removing Drugs	24
11	Bohe (BH)	Menthae Herba	Pungent Cool Diaphoretics	22
12	Zisu (SY)	Perilla Frutescens	Pungent-Warm Exterior-Releasing Medicinal	16
13	Jiegeng (JG)	Platycodon Grandiforus	Phlegresolving Medicine	14
14	Heye (HY)	Folium Nelumbinis	Antipyretic Detoxicate Drugs	14
15	Shengjiang (SJ)	Zingiber Officinale Roscoe	Pungent-Warm Exterior-Releasing Medicinal	13
16	Guanzhong (GZ)	Fortunes Bossfern Rhizome	Antipyretic Detoxicate Drugs	12
17	Fuling (FL)	Poria Cocos(Schw.) Wolf.	Diuretic Dampness Excreting Drugs	12

#### 3.1.2. Association and clustering analysis of high-frequency drugs in COVID-19 prevention and treatment formulas.

**Figure 1.**
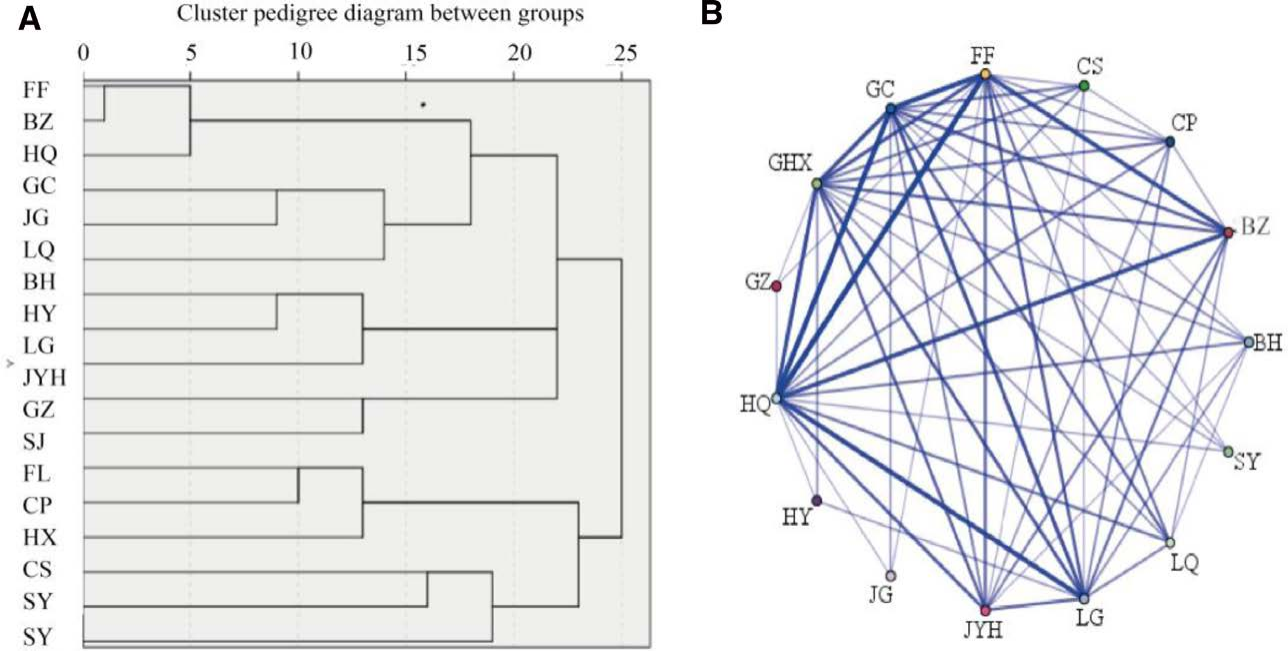
Clustering and association of frequently used drugs in COVID-19 prevention and treatment formulas. A. Hierarchical cluster dendrogram based on distance correlation. B. Association network diagram, where thicker and darker lines indicate higher co-occurrence probabilities. COVID-19 = coronavirus disease 2019.

The association analysis of drugs was performed based on the Apriori algorithm, and the top 3 drug pairs and drug groups were selected based on “support” and “confidence” (Table 2). The drug pair with the highest two-item association was “HQ + FF,” the top 3 supported drug pairs were HQ, FF, and BZ. Among the 3 associations, the “HQ + FF + BZ” combination ranked third in association degree. The association network diagram showed that HQ, FF, and BZ had strong correlations, and the cluster dendrogram showed that HQ, FF, and BZ were clustered together when the distance was 10 (Fig. 1). Based on these findings, “HQ, FF, BZ” (HFB) were selected as the core drug combination for preventing and treating COVID-19 for further research.

**Table 2:** Drug combinations in COVID-19 prevention and treatment formulas.

Herbal combination	Example	Support (%)	Confidence (%)
HQ + FF	44	50.00	84.09
FF + BZ	34	38.64	82.35
HQ + BZ	34	38.64	82.35
FF + GC + HQ	35	39.77	80.00
HQ + FF + GC	32	36.36	87.50
HQ + BZ + FF	28	31.82	92.86

### 3.2. Acquisition of effective ingredients and corresponding targets of HFB

Information on the effective ingredients of HFB was obtained from different databases, resulting in 72 effective ingredients and 467 targets after removing duplicates.

### 3.3. Acquisition of COVID-19-related targets

415 COVID-19-related targets were obtained from various databases, including 26 from DrugBank, 55 from Genecard, 103 from the Therapeutic Target Database, and 307 from DisGeNET after merging and removing duplicates.

### 3.4. Construction of HFB-active ingredients-target network

**Figure 2.**
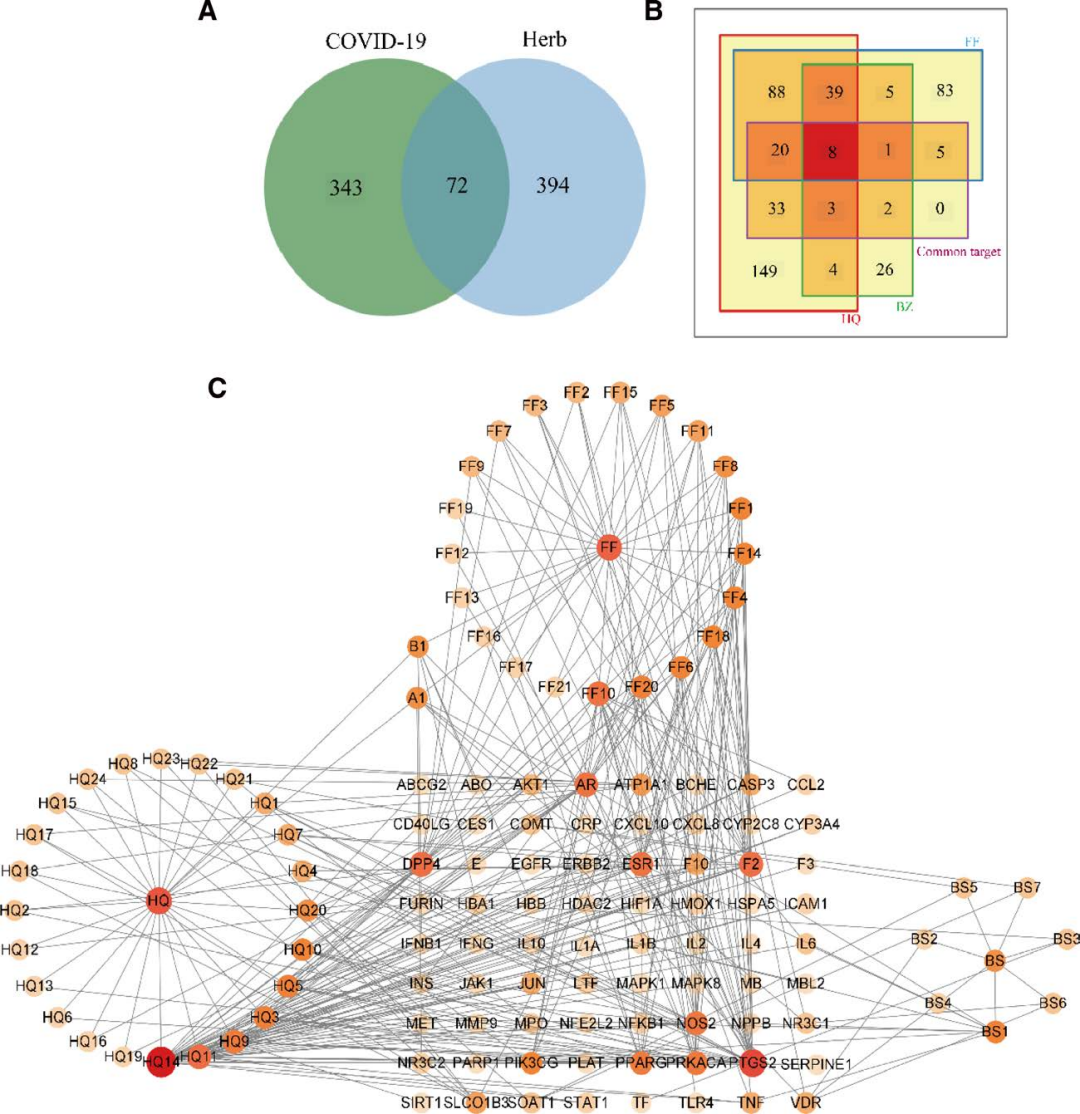
Network of HFB (Herbal Formula for COVID-19) active ingredients and targets. A. Venn diagram showing all targets of HFB and COVID-19-related targets. B. Square Venn diagram showing the targets corresponding to single herbal medicines in HFB and COVID-19-related targets. C. Network diagram of shared targets between HFB and COVID-19. COVID-19 = coronavirus disease 2019, HFB = HQ+FF+BZ.

The Venn diagram of the shared targets between HFB and COVID-19-related targets was created using the event website (http://www.bioinformatics.com.cn/static/others/jvenn/).^[33]^ There were 72 common targets between HFB and COVID-19 (Fig. 2A). Among these common targets, HQ corresponded to 64, FF to 34, and BZ to 14 targets (Fig. 2B). The HFB-Active Ingredients-Target network diagram was constructed using Cytoscape v3.9.0, showing 129 nodes and 316 edges (Fig. 2C).

### 3.5. Protein-protein interaction (PPI) network analysis

**Figure 3.**
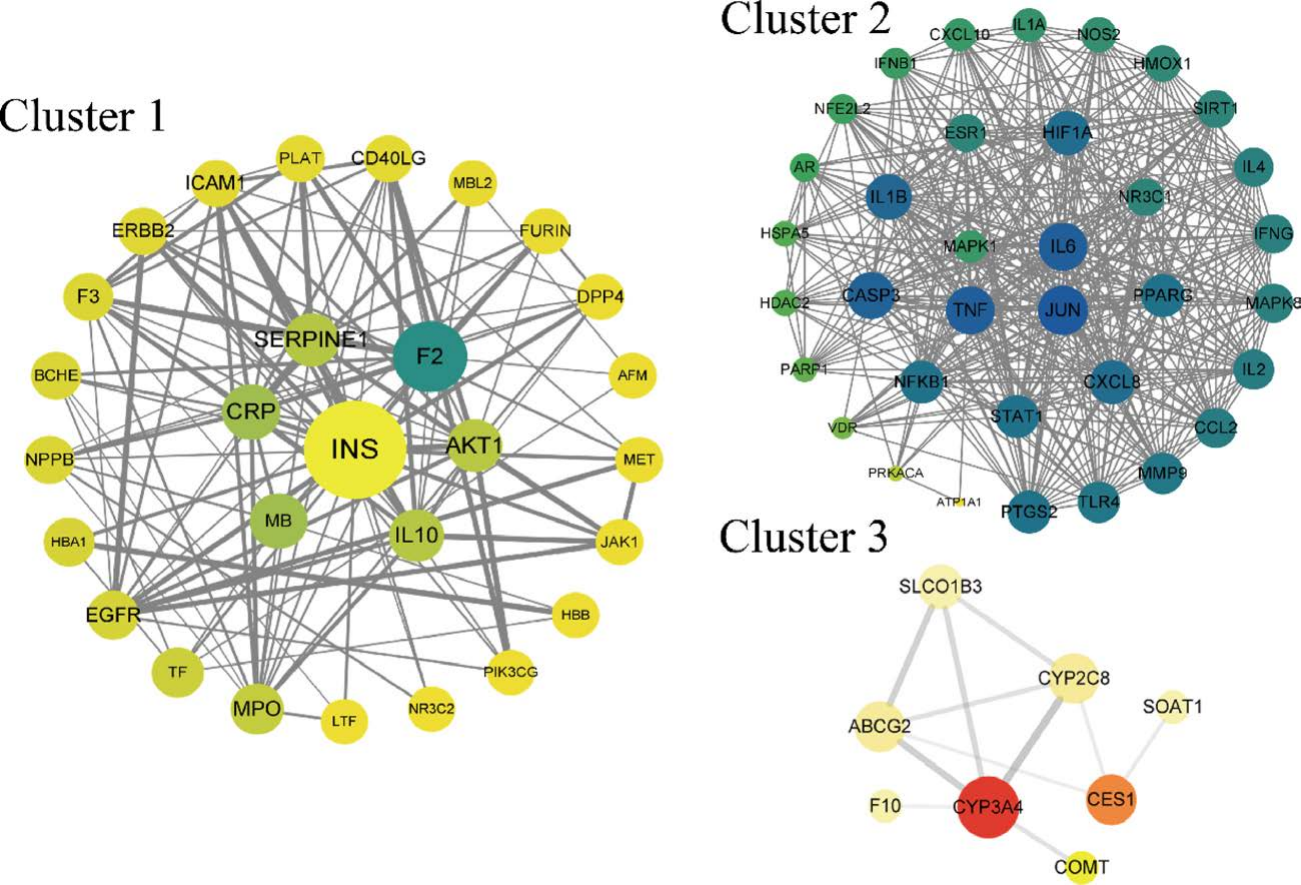
PPI network of shared targets between HFB (Herbal Formula for COVID-19) and COVID-19, and its clustered network diagram. Node size increases with higher betweenness centrality (BC) and closeness centrality (CC) values, while link thickness increases with higher correlation scores. COVID-19 = coronavirus disease 2019, HFB = HQ+FF+BZ.

The whole PPI network was clustered into 3 groups. Cluster 1, Cluster 2, and Cluster 3 contained 28, 36, and 8 targets, respectively. The values of degree (DC), betweenness (BC), and CC for each target were calculated using Cytoscape software, and the targets were arranged based on these values. Targets with BC and DC values below the mean were arranged in a circular layout, while those above the mean were arranged in a circular or rectangular layout inside the former. The thickness of the connecting lines was adjusted based on the combined score, with higher scores resulting in thicker lines (Fig. 3). High-scoring targets in Cluster 1 included INS, F2, IL10, SREPINE1, AKT1, etc. In Cluster 2, IL6, TNF, JUN, and MAPK1 had higher BC values. Cluster 3 had fewer enriched targets, with CYP3A4 showing significant enrichment. Therefore, INS, F2, IL10, SREPINE1, AKT1, IL6, TNF, JUN, MAPK1, and CYP3A4 were identified as potential key targets for preventing and treating COVID-19.

### 3.6. Analysis of gene expression differences

**Figure 4.**
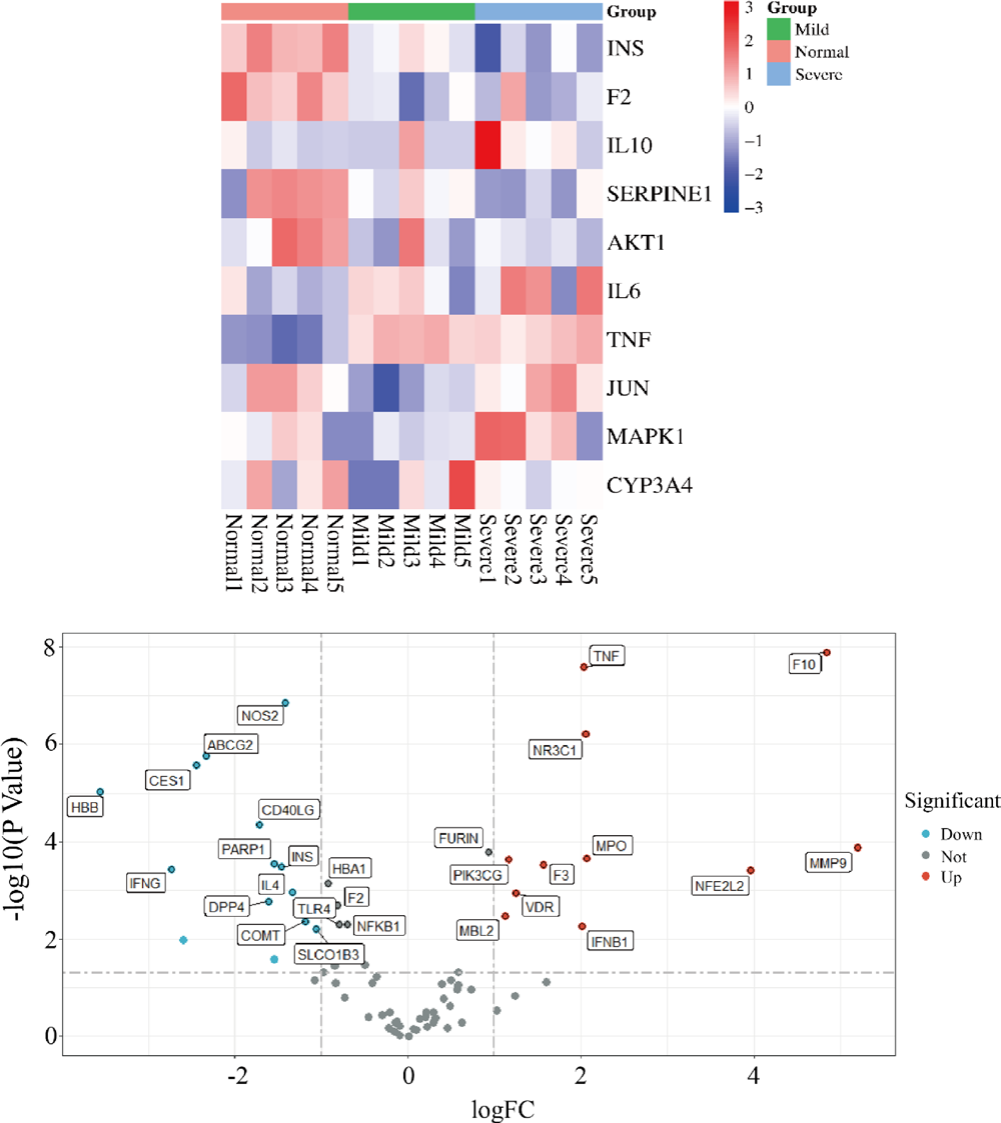
Expression of shared targets and core targets in GSE164805. The figures provided above showcase a core target gene expression heatmap and a volcano plot, demonstrating the expression levels of all shared target genes. Upregulated and downregulated genes are denoted by distinct color markers, enabling easy discrimination between the two.

Gene expression matrices were extracted from the GSE164805 gene chip data after annotation. The expression information of key targets was extracted from this matrix to analyze their expression changes in COVID-19. TNF and IL6 showed significant upregulation in COVID-19 patients, while the expression changes of the other targets were relatively minor (Fig. 4).

### 3.7. Gene ontology (GO) and Kyoto encyclopedia of genes and genomes (KEGG) enrichment analysis

**Figure 5.**
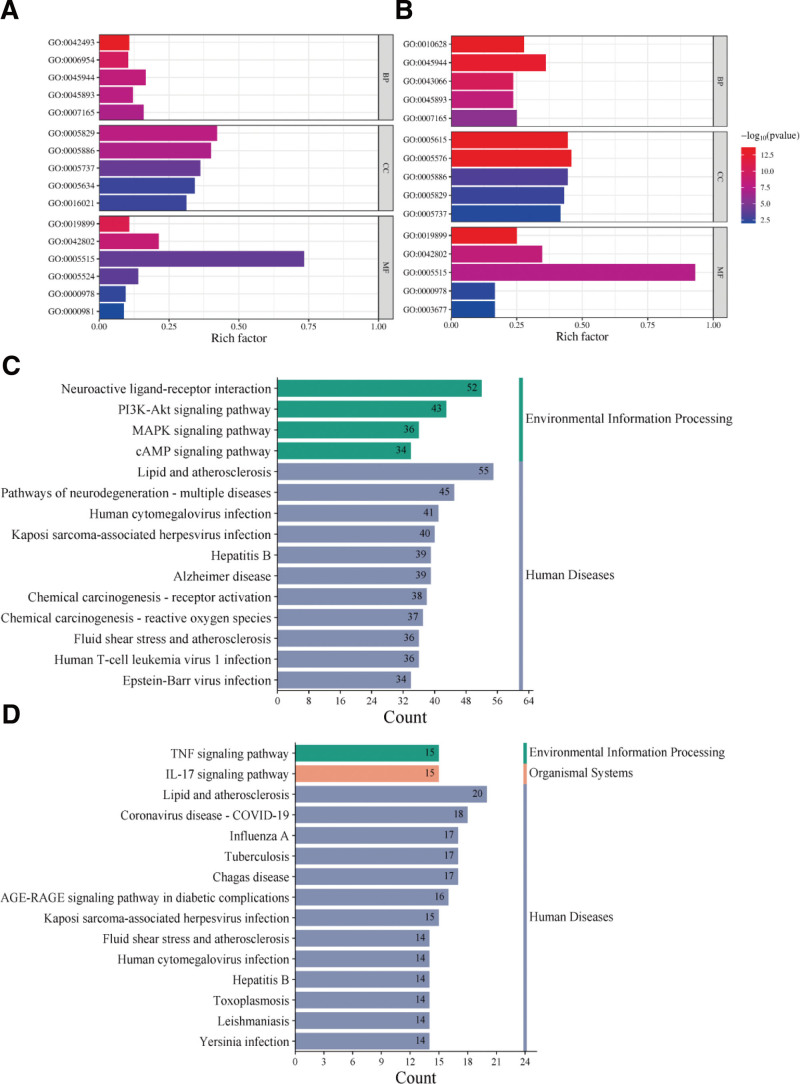
GO and KEGG analysis. A. GO analysis of all targets corresponding to effective compounds. B. GO analysis of shared targets. C. KEGG enrichment analysis of all targets corresponding to effective compounds. D. KEGG enrichment analysis of shared targets.

The analysis of 466 targets corresponding to HFB ingredients in the DAVID website revealed 496 BP items, 137 MF items, and 105 CC items with enriched gene numbers ≥ 5. We selected the 5 BP, CC, and MF entries with high enrichment for visualization (Fig. 5A). The MF items with relatively high enrichment gene numbers included RNA polymerase, 2 regulation-related items (GO:0000978, GO:0000981), and protein binding. The top enriched BP item was “positive regulation of transcription from RNA polymerase II promoter” (GO:0045944), and other enhanced items included “signal transduction” (GO:0007165). These target proteins were mainly distributed in the cytosol, plasma membrane, and cytoplasm (GO:0005829, GO:0005886, GO:0005737) (Fig. 5A).

In the GO analysis of common targets, there were 56 BP items, 32 MF items, and 25 CC items with enriched gene numbers ≥ 5. The most enhanced BP item was also “positive regulation of transcription from RNA polymerase II promoter.” The MF items with relatively high enrichment gene numbers included the GO:0000978 item, and there were also more genes enriched in the “cytokine activity” item (GO:0005125). These target proteins were mainly in the extracellular region (GO:0005576) (Fig. 5B).

The KEGG enrichment analysis revealed that the most enriched disease pathways for all targets corresponding to HFB ingredients included neuroactive ligand-receptor interaction pathways, lipid and atherosclerosis pathways, and pathways related to neurodegenerative diseases. The most enriched pathways for common targets included “Coronavirus disease - COVID-19” and “Influenza A pathway,” and the most enriched pathways in the Environmental Information Processing category were “TNF signaling pathway” (Fig. 5C and D).

**Figure 6.**
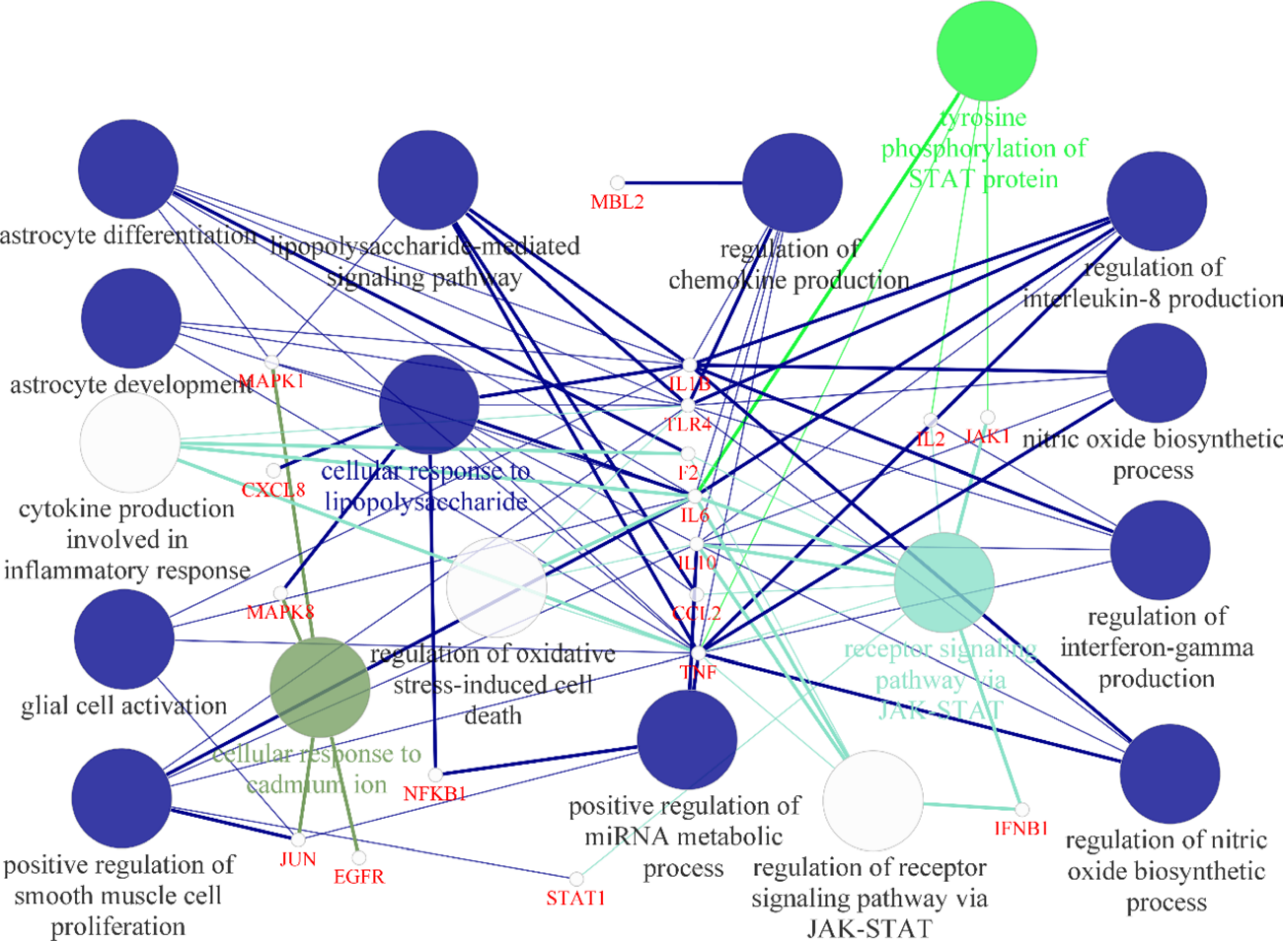
Network diagram of COVID-19-related pathway gene targets enriched by BF. COVID-19 = coronavirus disease 2019.

In the “Coronavirus disease - COVID-19” pathway, the enriched genes included JUN, CXCL8, IFNB1, STAT1, F2, TNF, EGFR, IL2, NFKB1, IL10, IL6, MAPK8, IL1B, CCL2, MAPK1, TLR4, JAK1, MBL2. These genes were mainly enriched in the “Tyrosine phosphorylation of STAT protein,” “receptor signaling pathway via JAK-STAT,” “cellular response to lipopolysaccharide,” and other BP items, and IL6, IL10, and TNF were involved in more molecular functions compared to other genes (Fig. 6).

### 3.8. Molecular docking of active ingredients and corresponding targets

The key targets in the PPI network also present in the “Coronavirus disease - COVID-19” pathway included IL6, MAPK1, JUN, F2, and TNF. The anti-inflammatory cytokine IL10 was strongly associated with IL6 and TNF in the PPI network,^[34,35]^ and IL6 and IL10 were critical factors related to fatal COVID-19.^[36]^ GO and KEGG analyses indicated that TNF, IL6, and IL10 played essential roles in the COVID-19 signaling pathway (Fig. 6). As a pro-inflammatory factor, TNF can affect the production of IL6 and other cytokines through the TNF signaling pathway (Figure S1, Supplemental Digital Content, http://links.lww.com/MD/K859). TNF was significantly upregulated in patients and is commonly used to construct cellular inflammation models,^[37,38]^ making it a potential drug for simulating the inflammatory phenomena caused by COVID-19. F2, which mediates the lectin pathway in the “Coronavirus disease – COVID-19” pathway (Figure S1, Supplemental Digital Content, http://links.lww.com/MD/K859), is often associated with coagulation disorders in severe COVID-19 patients.^[39,40]^ Anticoagulants are commonly used for severe patients,^[41]^ but their relevance to prevention and treatment is relatively low.

**Figure 7.**
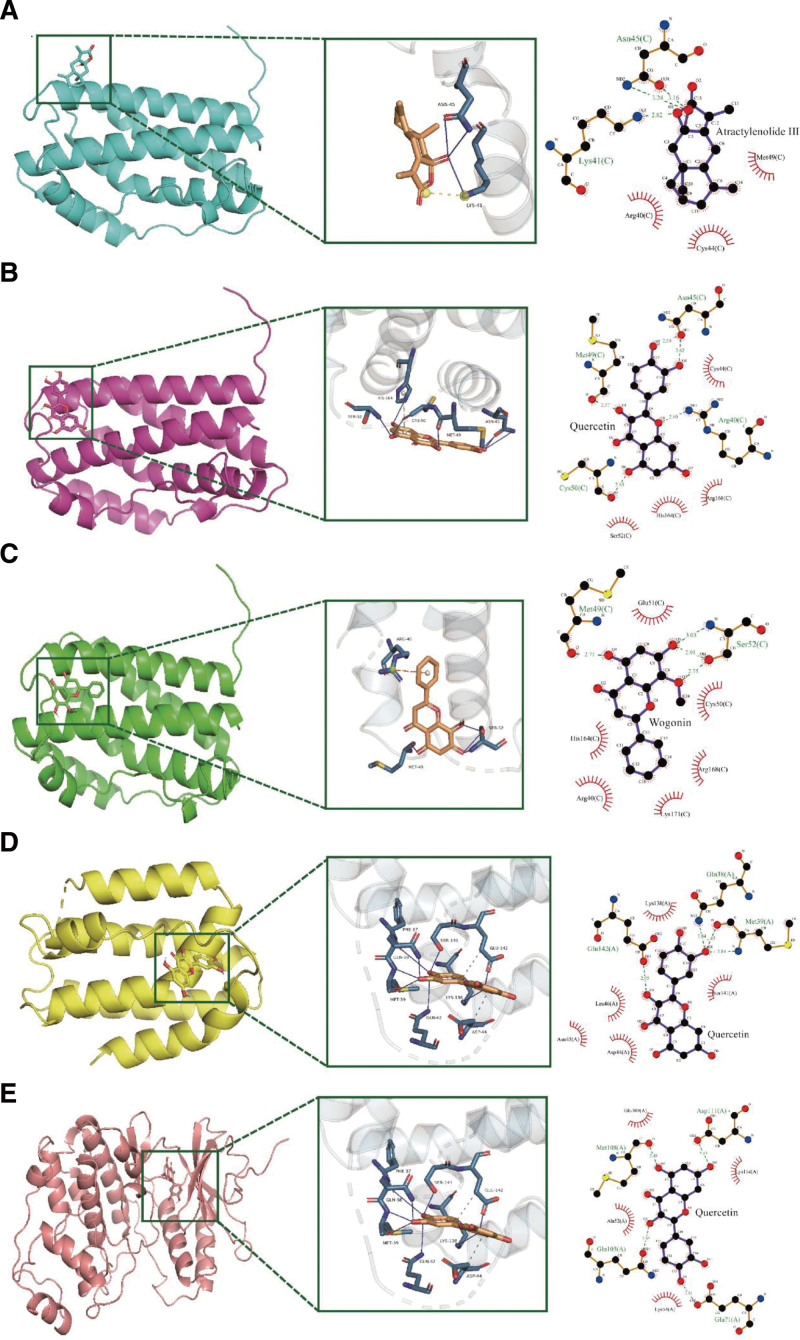
Docking conformations of HBF (Herbal Formula for COVID-19) corresponding targets. A. IL6 and Atractylenolide III. B. IL6 and quercetin. C. IL6 and wogonin. D. IL10 and quercetin. E. MAPK1 and quercetin. COVID-19 = coronavirus disease 2019, HFB = HQ+FF+BZ.

Quercetin corresponded to IL6, IL10, and MAPK1, while wogonin and Atractylenolide III corresponded to IL6. Molecular docking was performed to validate these 3 compounds and 3 targets. Generally, a docking score < 0 indicates possible binding, and a score < 4.25 kcal/mol indicates binding activity,^[42]^ with lower scores indicating more stable binding between ligands and proteins. Among all docking results, IL6 and quercetin showed the lowest docking scores, followed by Atractylenolide III (Table 3). The docking conformation of IL6 and quercetin suggested the possibility of π-Cation Interaction and multiple hydrogen bond connections. The docking conformation of IL10 and quercetin showed more hydrophobic interactions and hydrogen bond connections. MAPK1, quercetin, IL6, and wogonin had relatively high docking scores (Fig. 7).

**Table 3:** Binding energies of effective compounds and their corresponding targets.

Ingredient	Target	Affinity (kcal/mol)
Quercetin	IL6	−4.94
Quercetin	IL10	−4.73
Quercetin	MAPK1	−4.18
Atractylenolide III	IL6	−4.43
Wogonin	IL6	−3.9

### 3.9. In vitro experimental verification

**Figure 8.**
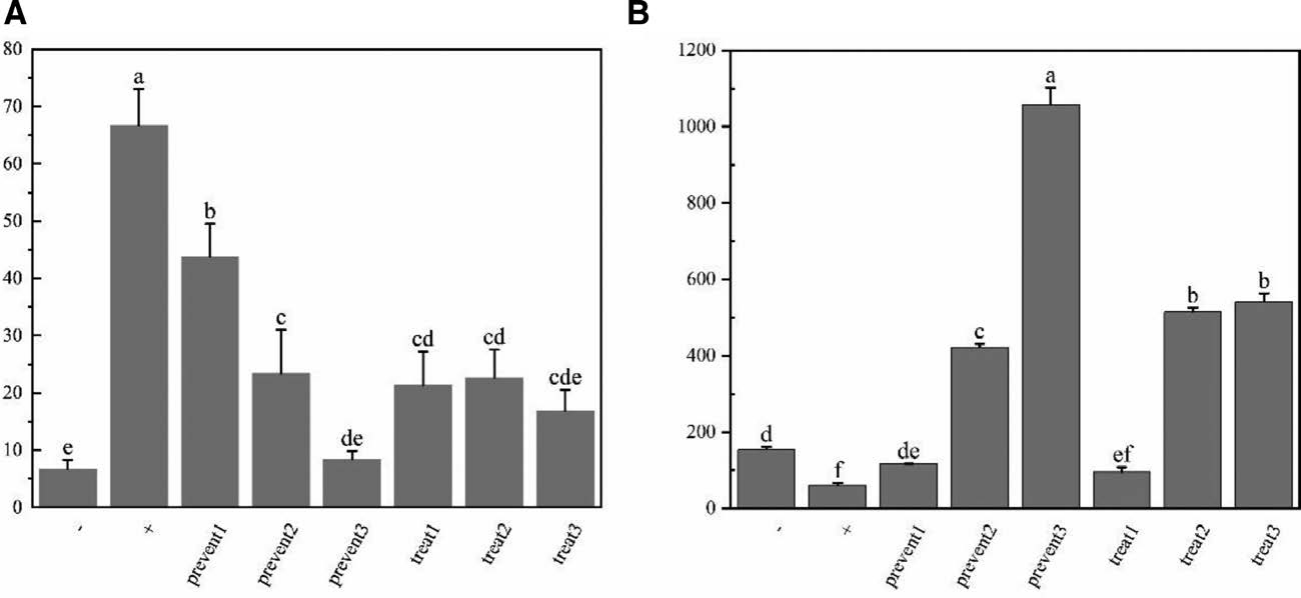
ELISA experimental results are indicated by different letters to denote significant differences. A. Levels of IL6 in each experimental group. B. Levels of IL10 in each experimental group. −: negative group; +: positive group.

Different experimental groups used other drugs, as shown in Table 4. The ELISA results showed that IL6 levels were significantly increased in the positive group compared to the negative group. With the increase in the concentration of quercetin, IL6 levels showed a decreasing trend and were not significantly different from the negative group at the highest quercetin concentration. IL6 levels did not differ significantly between different concentrations in the treatment group but were lower than those in the positive group. IL10 levels showed a slight decrease in the positive group but increased dramatically with the addition of quercetin in the prevention group. IL10 levels also increased significantly in the treatment group at high concentrations but did not show significant differences with the increase in concentration at high levels (Fig. 8).

**Table 4:** Use of drugs in each experimental group.

	−	+	Prevent 1	Prevent 2	Prevent 3	Treat 1	Treat 2	Treat 3
TNF-α (ng/mL)	0	10	10	10	10	10	10	10
quercetin (μg/mL)	0	0	1	5	10	1	5	10

−: No drug used, **+:** drug used.

## 4. Discussion

The cluster and association analysis showed that HQ, FF, and BZ were the core components of traditional Chinese medicines for COVID-19 prevention and control. This combination is consistent with the formula of the traditional Chinese herbal medicine “Yupingfeng San.” “Yupingfeng San” is widely used in respiratory system diseases and has demonstrated sound auxiliary therapeutic effects in combination with Western medicine for respiratory system diseases. Wei et al^[43]^ found that the combination of “Yupingfeng San” and montelukast sodium significantly reduced the levels of inflammatory factors and improved disease symptoms in children during the remission phase of asthma without any adverse reactions. Zhang et al^[44]^ found that azithromycin and “Yupingfeng San” particles could alleviate cough, high fever, and other symptoms in children with mycoplasma pneumonia and reduce C-reactive protein and white blood cell counts. In cases of COVID-19, the combination of “Xiao Chaihu Tang” and “Yupingfeng San” can effectively shorten the course of COVID-19 and improve fever and other symptoms.^[45]^ Additionally, the clinical efficacy of “Yupingfeng San” in treating children with disharmony between the nutrient and defensive aspects of the body (biao xu zi han) was observed. Treatment with “Yupingfeng San” led to increased IgG, IgM, IgA, C3, and C4 levels and decreased upper respiratory tract infections. These improvements in Biao xu zi han symptoms indicate enhanced immune function and suggest its potential as a drug for preventing and treating COVID-19.^[46]^ The combination of BZ, HQ, and FF holds promise for preventing and treating COVID-19.

Quercetin is the ingredient that corresponds to the most COVID-19-related targets among the 72 active ingredients in the HQ, FF, and BZ drug groups. Quercetin is widely distributed in Chinese herbs; related research has been carried out relatively early, resulting in numerous corresponding target components. Quercetin and its derivatives have demonstrated extensive pharmacological effects, including anti-inflammatory, antiviral, and antioxidant activities.^[47]^ Previous studies have shown that quercetin supplementation can significantly reduce inflammatory factors such as IL-6 and TNF-α expression in Zucker diabetic fatty rats with type 2 diabetes.^[48]^ In a lipopolysaccharide-induced colitis mouse model, quercetin improved intestinal inflammation effectively caused by lipopolysaccharide and reduced the expression levels of inflammatory factors IL-6 and IL-12.^[49]^

The ultimate goal of clustering is to make targets within the group similar, while there are differences in the objects between different groups.^[50]^ K-means clustering uses Euclidean distance as the similarity measure and the sum of squared errors as the objective function to measure the clustering quality. Minimizing the objective function divides data points into k clusters according to the distance from the cluster center.^[51]^ According to the comprehensive scores given by the STRING website, the PPI network of the original 72 common targets was clustered into 3 separate similar clusters using K-means clustering, and core targets were then identified in each of these clusters based on BC, DC, and CC values. Through this method, the core targets of HFB were screened out as INS, F2, IL10, SREPINE1, AKT1, IL6, TNF, JUN, MAPK1, and CYP3A4.

Among all the enriched BP entries corresponding to HFB components and common target proteins, there are 8 identical ones, including “Positive regulation of RNA polymerase II promoter transcription” and “Inflammatory response (GO:0006954).” The BP entries of common targets also included “cellular response to lipopolysaccharide (GO:0071222)” and “negative regulation of transcription from RNA polymerase II promoter (GO:0000122)” (Fig. 6A and B). In addition, compared to all targets, the CC entries enriched by common targets were more in the extracellular region. In the KEGG analysis of common targets, more genes were enriched in pro-inflammatory cytokine pathways, such as the TNF and IL-17 signaling pathways. The genes involved in COVID-19 mainly participate in biological processes such as the JAK-STAT receptor signaling pathway. It is possible that their regulation of COVID-19 may be related to this pathway.

After entering cells through ACE2, SARS-CoV-2 relies on transmembrane serine protease 2 (TMPRSS2) and endosomal cysteine proteases cathepsin B and L (CatB/L) to initiate infection.^[52]^ Followed by the occupation of ACE2 by SARS-CoV-2 is the increase of its original substrate, angiotensin II, which promotes the activation of the NF-κB pathway and results in the production of inflammatory cytokines such as IL-6 and TNF (Figure S1, Supplemental Digital Content, http://links.lww.com/MD/K859).^[53]^ At the same time, angiotensin II activates ADAM17 protease activity, further leading to the production of TNF-α, HBEGF, and IL-6Rα, activating the NF-κB pathway and IL-6 trans-signaling. This is known as the IL-6 amplifier (IL6-AMP) (Figure S1, Supplemental Digital Content, http://links.lww.com/MD/K859).^[53]^ From this perspective, TNF promotes the release of inflammatory cytokines such as IL-6. In addition, in TNF-α-induced HaCaT cells, it was found to significantly increase the mRNA expression levels of pro-inflammatory factors such as IL-6.^[54]^

Molecular docking shows quercetin has good docking results with IL-6 and IL-10, suggesting possible binding. The A549 alveolar epithelial cells are commonly used as a cell model, and their characteristics as lung cells make them more suitable for the subject of this experiment. By utilizing TNF-α to establish the corresponding model, the early infection-induced inflammation was created to evaluate quercetin’s preventive and therapeutic effects on inflammation. The results showed that in the preventive and therapeutic groups with both quercetin and TNF-α, the expression of IL-6 was effectively reduced relative to the positive group, and the expression of the anti-inflammatory factor IL-10 was increased. In the treatment group where quercetin was added later, IL-6 levels decreased relative to the positive group, but there was no significant difference with the change in quercetin dosage. In the high-dose quercetin treatment group, IL-10 levels were significantly increased relative to the positive group.

In summary, this study combined data mining, network pharmacology, molecular docking, and other bioinformatics methods to explore the information on COVID-19 prevention and treatment prescriptions from various regions, identify key drug combinations, and investigate their mechanisms for preventing and treating COVID-19. The results showed that the core herbal formula was composed of “HQ, BZ, FF,” consistent with the traditional formula “Yupingfeng San.” This drug combination may affect the progression of COVID-19 through inflammatory pathways such as the TNF signaling pathway. Further PPI network analysis, differential analysis, and molecular docking revealed that the critical component quercetin affects the inflammatory response caused by COVID-19 through the core targets IL-6 and IL-10. In vitro experiments demonstrated that quercetin effectively reduced the levels of the inflammatory factor IL-6 and increased the anti-inflammatory factor IL-10 in the preventive and therapeutic groups of a TNF-induced pulmonary cell inflammation model, thereby alleviating the impact of inflammation on cells. This study provides a new idea for understanding the molecular mechanism of COVID-19 prevention and provides some support for the further use of herbal compounding to prevent COVID-19.

## Author contributions

**Conceptualization:** Jiakai Yang.

**Data curation:** Jiakai Yang, Chi Zhang.

**Formal analysis:** Jiakai Yang.

**Funding acquisition:** Qianqian Zhuang.

**Project administration:** Xinli Liu.

**Resources:** Jiakai Yang.

**Software:** Jiakai Yang.

**Supervision:** Xinli Liu.

**Validation:** Jiakai Yang.

**Visualization:** Jiakai Yang.

**Writing – original draft:** Jiakai Yang.

**Writing – review & editing:** Jiakai Yang.

## Supplementary Material


