# Supplementary material for: Active ingredient and mechanistic analysis of traditional Chinese medicine formulas for the prevention and treatment of COVID-19: Insights from bioinformatics and in vitro experiments
Source: Medicine (Baltimore). 2023 Dec 1;102(48):e36238. doi: 10.1097/MD.0000000000036238 (PMC10695544; doi:10.1097/MD.0000000000036238)
Supplement: Supplementary file 1 [file medi-102-e36238-s001.docx]

、
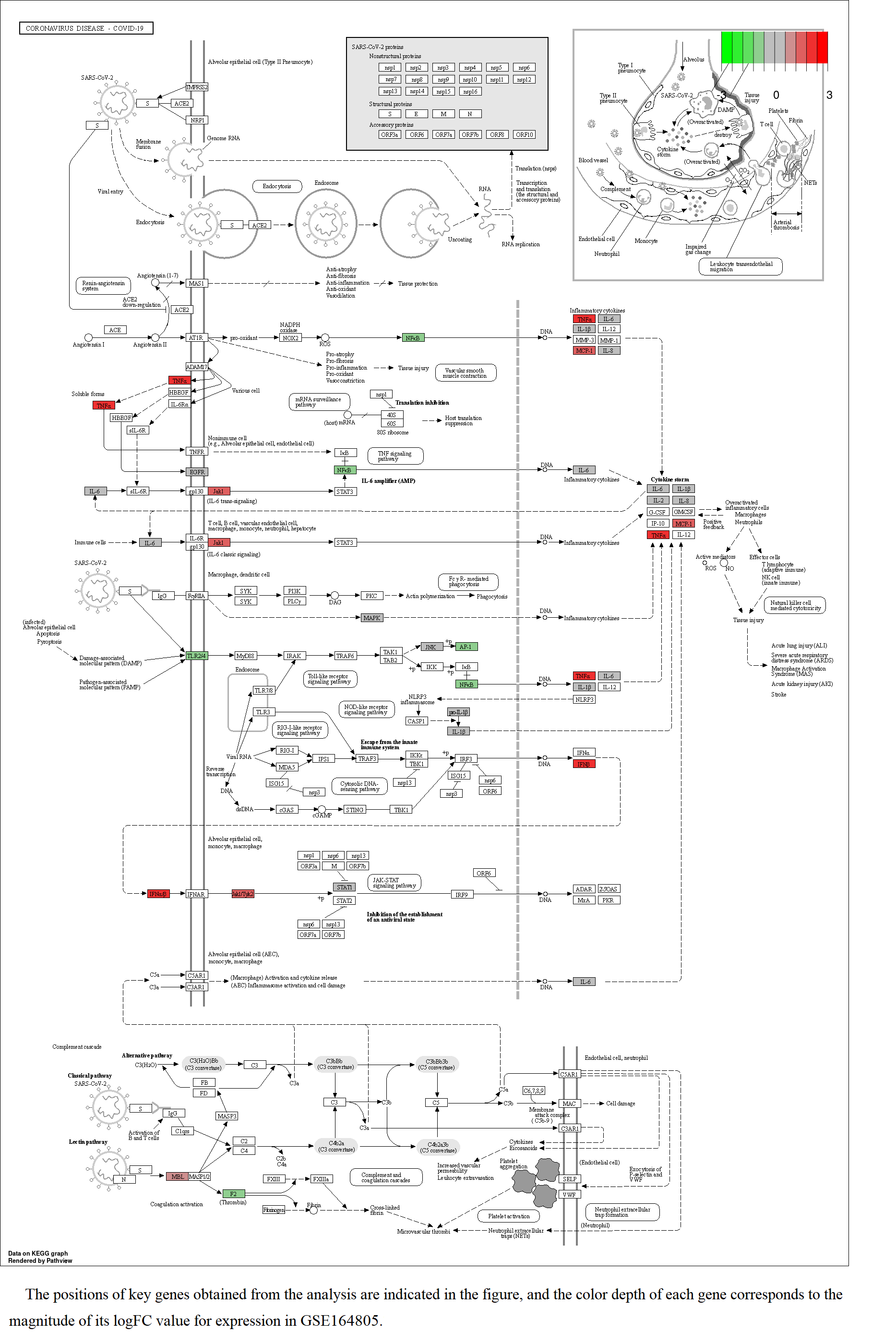


**Supplementary figure 1.** The positions of key genes obtained from the analysis are indicated in the figure, and the color depth of each gene corresponds to the magnitude of its logFC value for expression in GSE164805.
